# Revision of a Monoblock Metal-on-Metal Cup Using a Dual Mobility Component: Is It a Reasonable Option?

**DOI:** 10.3390/ma13092040

**Published:** 2020-04-27

**Authors:** Saverio Affatato, Emanuela Castiello, Luca Amendola, Saverio Comitini, Jean Louis Prudhon, Domenico Tigani

**Affiliations:** 1 Laboratorio di Tecnologia Medica, IRCCS – Istituto Ortopedico Rizzoli, 40100 Bologna, Italy; 2 Unità Operativa Complessa di Ortopedia e Traumatologia, Ospedale Maggiore, 40100 Bologna, Italy; emanuela.castiello@ausl.bologna.it (E.C.); luca.amendola@ausl.bo.it (L.A.); saverio.comitini@ausl.bo.it (S.C.); domenico.tigani@ausl.bologna.it (D.T.); 3 Centre Osteo-Articulaire Echirolles, 38100 Grenoble, France; jean-louis.prudhon@wanadoo.fr

**Keywords:** revision, MoM, dual mobility, hip, total hip arthroplasty, off label use

## Abstract

Revision of large-diameter, monoblock acetabular components for both hip resurfacing arthroplasty and metal-on-metal (MoM) total hip arthroplasty (THA) is correlated to a high amount of complications. For this reason, performing a limited revision by conversion to a dual mobility (DM) without acetabular component exchange has been proposed in order to limit these complications. Although DM bearing offers an easy solution avoiding the intraoperative and time-associated complications, concern about polyethylene wear and stability remains due to the difference regarding the design, the coverage angle and the clearance of the two implants. In order to evaluate the performance of this new solution with the new material to prevent the possibility of failure it is essential to conduct a review of the literature A qualitative systematic review of the literature has been conducted according to the Preferred Reporting Items for Systematic Reviews and Meta-Analyses guidelines. A comprehensive search of PubMed, EMBASE, Google Scholar, and Scopus for English and French articles between January 2000 and October 2019 was performed, with the primary objective of finding articles about dual mobility bearing coupling with large metal-on-metal cup in the case of hip revision procedure. Various combinations of the key words were used in the search strategy. Thirteen articles with DM bearing mated with MoM cup were analyzed. Of the 130 hip revisions selected, with a follow-up from 6 to 53 months, there were a total of 14 with complications (10.77%): four true dislocations (3.08%); six intra-prosthetic dislocations (IPD, 4.6%), two of which presented plastic deformation and polyethylene wear; four other complications (3.08%), included a cup osteolysis, a clicking noise, a superficial infection and a periprosthetic fracture. All the mentioned true dislocations occurred during the first month while IPDs appeared during the first two years from the index revision. In conclusion, according to the literature analyzed, we can stress that the concerns and doubts about mating a DM bearing with large MoM cup cannot be dissolved. It has been pointed out that a DM bearing is not designed for a MoM cup; it is not mechanically tested on MoM cups, which presents different clearance and coverage angles. Predictable complications may occur, such as IPD, polyethylene wear and true dislocation. These complications have been reported at an even higher rate than they were in the eighties, when the first generation of DM implants were of a lower quality of polyethylene and the characteristic of the design was less optimal than modern ones.

## 1. Introduction

Hip resurfacing arthroplasty (HRA) and metal-on-metal (MoM) total hip arthroplasty implants have been advocated as an option for the treatment of degenerative hip disease for young and active patients [1]. Metal-on-metal (MoM) bearings have been used since early on in the age of modern total hip arthroplasty (THA) [2] although this solution has been gradually replaced by the low friction concept of Sir John Charnley [3] and by other hard bearing surfaces like ceramic/ceramic which were first introduced by Boutin in France in the early seventies [4]. In the nineties a second generation of MoM-THR, including resurfacings, was introduced with the intention of reducing wear [5] and improving stability and range of motion. Although early reports were encouraging [6], subsequent joint registry data [7] and clinical studies [8,9,10] have highlighted the increased revision rates of metal-on-metal (MoM) and hip resurfacing arthroplasties (HRA). Elevated metal ion levels, corrosion that can lead to osteolysis, and local adverse soft tissue reactions like adverse reaction to metal debris (ARMD), as well as fracture of the femoral neck, are well-documented complications that need early revision surgery [11,12]. Revision of a large-diameter monoblock acetabular component in failed MoM hips remains a challenging procedure for the orthopaedic surgeon, especially in the presence of well-fixed and well-positioned components. Some authors reported that cup revision in MoM can be technically difficult [13], it is often associated with loss of bone stock and could increase the risk of dislocation when small heads are used for revision surgery [14]. Therefore, it has been proposed to leave in place the acetabular component and to convert the implant into a dual mobility system [13,15,16,17,18,19,20,21,22,23,24,25,26]. Although dual mobility (DM) bearings offer an easy solution avoiding the intraoperative and time-associated complications related to the removal of a well-fixed and well-positioned acetabular shell, other considerations should be evaluated before this new solution is considered as reliable and reproducible. Concerns remain due to the differences in the design between these two systems of hip arthroplasties as well as the biomechanical features of two different concepts between the Dual mobility cup and MoM implants. Therefore, it may be appropriated to evaluate the clinical performance of this new surgical solution in order to decide if there is a rationale in mating two different philosophies of design and material construction. 

We conducted a qualitative systematic review with the primary objective of analyzing the incidence of all the complications connected with the procedure and particularly the dislocation rate of the hips as well as the intra prosthetic dislocation (IPD); the latter is a specific complication of dual mobility, in which the femoral head separates from the liner (small articulation) due to the wear failure of the retentive mechanism between the mobile polyethylene and femoral head [27], observed with significant incidence in the first generation of this DM implant.

## 2. Materials and Methods

We conducted a qualitative systematic review of the literature according to the Preferred Reporting Items for Systematic Reviews and Meta-Analyses guidelines [28] in order to analyze the result of a limited revision of metal-on-metal hip arthroplasty compared to leaving in place the acetabular component and using a dual mobility bearing. 

Literary research was performed on all published and available reports in worldwide databases. In particular, we used available data from the following databases: PubMed, EMBASE, Google Scholar, Scopus. Patients were not involved in the conception, design, analysis, drafting, interpretation or revision of this research. Thus, ethics approval was not required. The inclusion criteria take into account all studies about hip revisions in which a dual mobility bearing has been mated to a monoblock acetabular component designed specifically for large-diameter metal-on-metal bearing. Exclusion criteria included studies with primary hip arthroplasties, revision surgeries for infection and revisions with small metal heads (less than 40 mm in diameter). Biomechanical studies, technical notes, letters to editors, literature reviews, and expert opinions were excluded. Reference lists of the included articles were carefully checked for missed studies. Both English and French clinical studies were evaluated. Key words used in the search strategy included “dual mobility,” “dual-mobility,” “tripolar”, “double mobility”, “double-mobility”, “Revision” “Metal on Metal”, “MoM”, “hip”, “cup”, and “socket”. All the prostheses that partially or completely matched these criteria were selected. We included all the articles published from January 2000 to October 2019.

Two independent reviewers (D.T. and S.A.) experienced in international literature, screened all titles and abstracts of all the articles in order to evaluate their inclusion, or exclusion, in this study. 

## 3. Results

**Figure 1:**
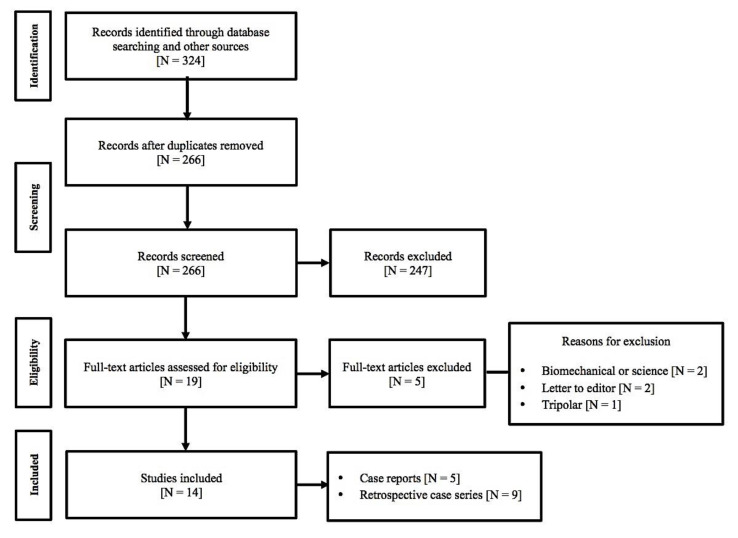
Flow chart showing our inclusion criterion.

Our international research found 324 potential articles. A critical selection process of all studies in which there was a correlation between MoM cup and DM was applied. A total of 14 articles have met our inclusion criterion (Figure 1—flow chart) for a total of 130 revision cases. 

Twenty-two patients received the Active Articulation DM E1 but there were only three cases of coupling with M2a-Magnum^®^cup (Biomet, Inc, Warsaw, IN, USA); this implant received Food & Drug Administration (FDA) clearance [29] as well as European conformity assessment [23,30]. The remaining 127 hips, were either Active Articulation DM E1 or ADM/MDM X3 (Stryker, Mahwah, NJ, USA), and were all off-label procedures where the outer diameter of the dual-mobility polyethylene bearing, of the same or often of a different company, was matched to the inner diameter of the retained shell. 

The inner femoral head materials used at the time of revision included metal, ceramic or ceramic coated metal of 22.2 or 28 mm according to the size of the liner. Major details are shown in Table 1.

Patients’ demographics were not reported in Table 1 because of the heterogeneity of patients. In the same way, we did not report clinical evaluation because it was conducted by different authors according to different scores, i.e., the Harry Hip Score (HHS), the Merle D’Aubignè hip score, the Western Ontario and Mac Master Universities Osteoarthritis (WOMAC) and the University of California, Los Angeles (UCLA) activity score

All patients treated in the selected studies were followed from 6 to 53 months. Regarding the complications occurred we stratified the cases according to three groups (Table 2): the first one included true dislocation, the second group incorporated all cases with intra-prosthetic dislocation (IPD), all other complications were included in the third group.

True dislocation (group 1) was reported in four patients during the first month from the index procedure. According to the liners implanted, two cases were related to the use of ADM/MDM X3^®^ (Stryker, Mahwah, NJ, USA) [13] and another with the Active Articulation DM E1^®^, and for the fourth case the liner was not mentioned as both types were used in the series [26]. A close reduction was performed in three cases: one of them had a partial dissociation of the head during close reduction and was revised with a new polyethylene liner and a new small head. In another patient the polyethylene liner has detached from the small femoral head because of the “bottle-opener” effect during limb traction maneuver for closed reduction; this case has been therefore revised with a new polyethylene liner and a new small head. The third patient had two dislocations managed with closed reductions. The dislocations occurred at 4 weeks postoperatively and at 3 months post-operatively during stretching with physical therapy. No other dislocations or surgical procedures were reported for this patient. The last patient was submitted to revision owing to recurrent instability during the first month after the index operation.

Five articles were included in group 2 due to the observation of IPD; there were three case reports [17,23,24] and two case series [20,25], for a total of six cases (4.6% of the total); two patients belonged to the same series [25]. Two articles documented the index complications associated with catastrophic deformations of polyethylene [23,24]. Two IPDs occurred during the first year, another two at the 14th and 19th months, respectively, the remaining two patients manifested IPD at 3.8 and 3.9 years from the index procedure.

Group 3, which contained all other complications, reported three cases of revisions. One patient showed sustained early infection treated by debridement with retention of the implant. Another case reported periprosthetic fracture, this patient underwent open fixation, revision of the femoral component and substitution of the DM head construct. The last patient had cup revision due to osteolysis. Finally, a case of a clicking noise was reported without pain and treated conservatively.

Radiological data were missing in many cases or were not accurate. When reported, failed implants have an average cup inclination and anteversion of 49° and 35°, respectively [25]. In five of the six patients who experienced IPD failure the abduction angles were beyond 45° (from 48 to 72°) [17,20,24].

## 4. Discussion

The results of our qualitative systematic review lead us to make some general observations regarding the manifestation of some observed complications, particularly about the true dislocation and the IPD.

The rationale of leaving the well-fixed acetabular component in situ and converting the implant to a dual mobility is certainly correct and starts from the assumption to reduce intra- and post-operative morbidity and to preserve bone stock, as documented by several reports [12,20,22,25,26]. Nevertheless, the incidence of so many cases of complications for a total of 10.77%, of which 10 (7.6%) of true dislocations and intra-prosthetic dislocations in a such short period of time, requires proper debate and consideration, especially considering that the majority of procedures are off-label practices, therefore unsupported by the Food and Drug Administration, the European International Medical Devices Regulators Forum and manufacturers. However, beyond the medico-legal implications, in our opinion, it is interesting to analyze the different construction philosophies of the two implants, MoM cup and dual mobility socket, to try to give an explanation of the occurred failures.

**Figure 2:**
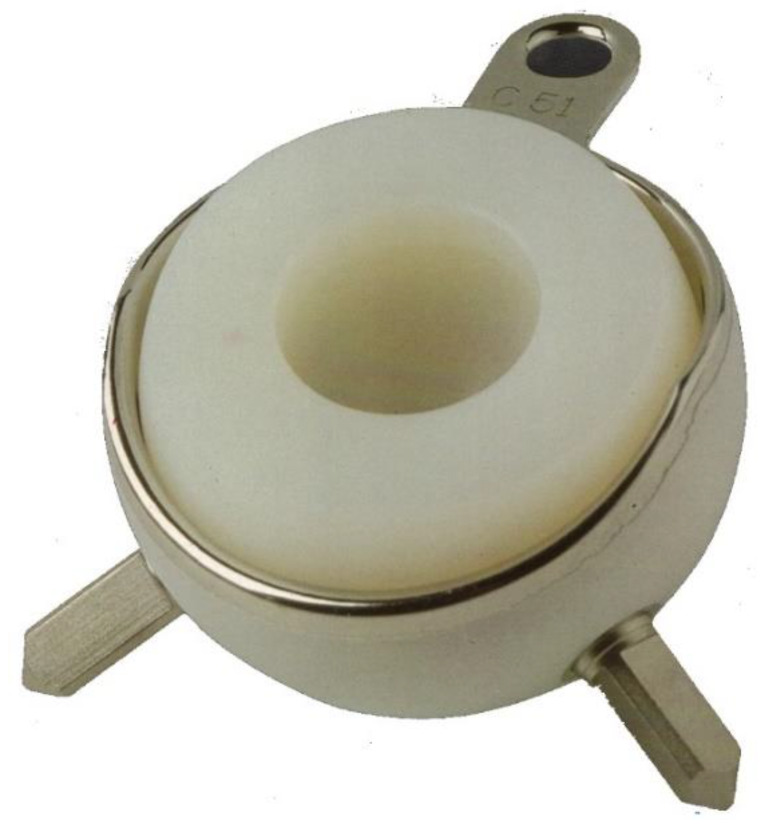
Original Bousquet design «tripode configuration». Courtesy of SER F, Décines, France.

The main design of DM cup is based on a “supra-hemispherical, cylindrical-shaped edge” at the roof [31]. In the original Bousquet design [32], precursor of the “hat design” and tripode configuration, the overhang of the hemisphere reached 8 mm for an area which largely went from the equator to the upper pole and the shape of “hat”, whereas in the modern implants the metallic shell may have three different aspects for the monoblock shell, which in the last decade has been associated with several modular implants, Figure 2.

**Figure 3:**
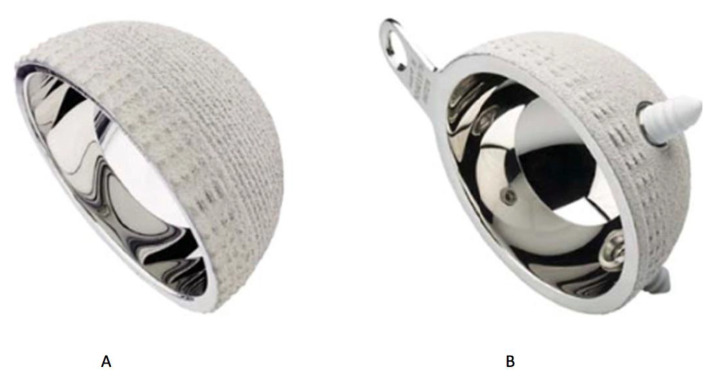
Part (**A**) shows the Sunfit SERF (cylindrospheric design) while part (**B**) shows the Novae E (Tripode configuration). Courtesy of SERF, Décines, France.

Moreover, the monoblock cups may be partitioned in: cylindrospheric design; modern “hat design” and anatomical design. The cylindrospheric design consisted of a half sphere augmented by a cylinder of 3 mm at the equatorial area (Figure 3).

**Figure 4:**
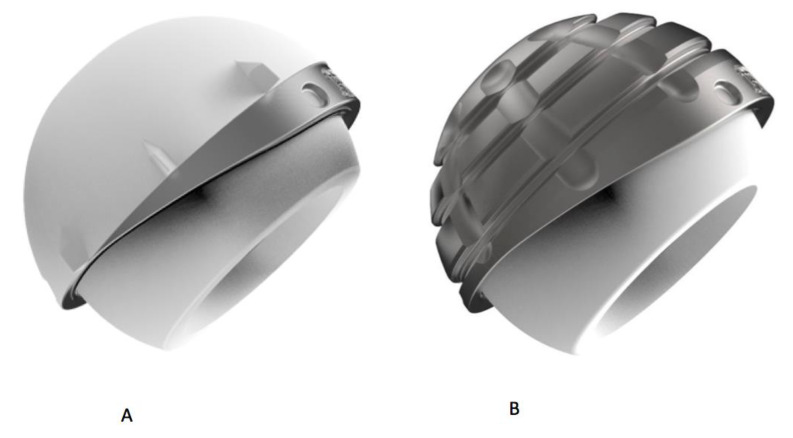
«Quattro» acetabular component with its “hat configuration” or “cupules à casquette”: part (**A**) shows the cementless component and part (**B**) the cemented one. Courtesy of Groupe Lépine, Genay, France.

The modern “hat designs” are characterized by the presence of an inclined plane, which increases the coverage angle until 12°. Often, the upper rim is covered in order to avoid polyethylene wear of the mobile liner and improve the stability, according to a so called “hat design” or “cupules à casquette”, (Figure 4) [31].

**Figure 5:**
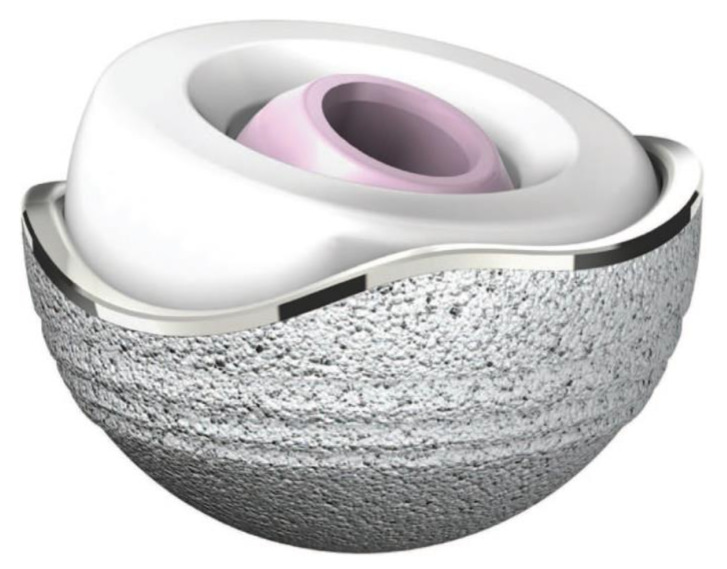
Anatomical design Dual-Mobility (ADM) X3 dual-mobility hip system. Courtesy of Stryker, Italia.

Finally, it is possible to have an anatomical design branded by an anatomic-shaped rim to match the native acetabular socket and accommodate the ileo-psoas tendon, and a posterior and inferior prominence, which augments the deepness of the shell [33], (Figure 5).

This is in contrast with almost all MoM cups that displaced less than the hemispheric shape [34], with a lower profile and a smaller inner surface design.

**Figure 6:**
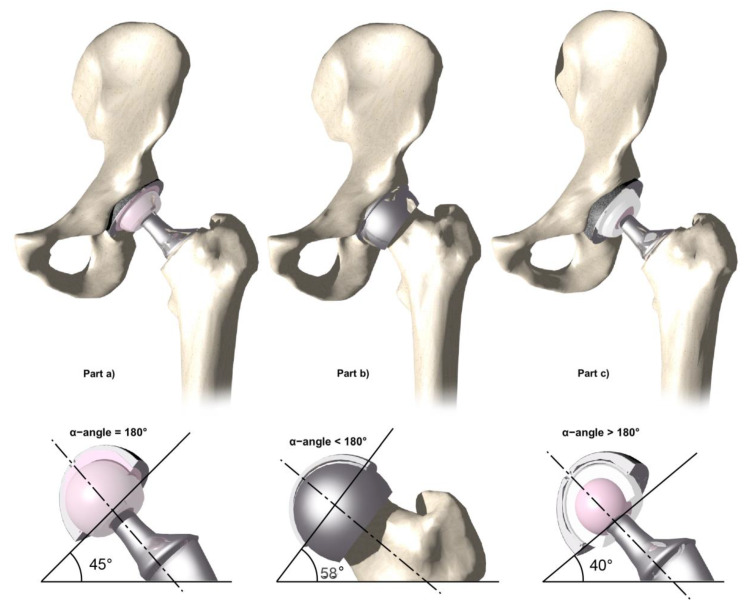
Effective inclination angles of various designs assuming an abduction angle of 45°. Part (**a**) shows a standard hip implant with an abduction angle of 45°(180° coverage angle); part (**b**) shows a Birmingham Hip Resurfacing (BHR) resurfacing hip implant with an effective angle of inclination of 58° (less than 180° coverage angle); part (**c**) shows a DM hip implant with an angle of inclination of 40° (more than 180° coverage angle).

This small inner surface determines the so-called α-angle, or coverage angle; this measure is specific to each acetabular component and it is the coverage of the socket on the femoral head [35] (Figure 6). The coverage of the femoral head decreases with the higher abduction of the cup (stepper positioning of the cup) or with the smaller size of the acetabular component. In the classical metal-on-plastic joint, the coverage angle is 180° or a full hemisphere (Figure 6—Part a). In this condition, the implant positioned at 45° of inclination, considered as “ideal”, is the real position of the socket. In MoM hip implants, the α−angle is less than 180° (Figure 6—Part b). In this configuration, because of its smaller coverage angle, 45° of abduction corresponds to a higher abduction of the cup according to the formula 180° − α angle/2 [35]. In Figure 6—Part c, the coverage angle greater than 180° is represented. Furthermore, different designs may produce different coverage angles according to the size of the implant.

In the case of a half sphere augmented by a cylinder of 3mm DM cup, the effective inclination is minus 5 to 7 degrees, according to the size [31]. On the contrary, in MoM cup the abduction angle is much higher than DM beta angle in relation to the coverage angle [36,37]. An important consequence of the parameters hardly mentioned is the reduction of jumping distance and therefore the major risk of dislocation. This situation is also accentuated by the fact that the femoral head center is outside the cup center. Viste et al. have calculated that the jumping distance is about 20% lower with a MoM cup compared with DM [38].

In order to understand the cause of wear and IPD it is important to verify if there is a difference in radial clearance between the MoM and the DM liner implanted. In fact, even if prosthetic hip joints are considered perfectly spherical, in reality the outer diameter of the femoral head is slightly smaller than the inner diameter of the acetabular liner, and therefore the components articulate with some eccentricity. This eccentricity is called “clearance” and it is important to ensure smooth tribology as it prevents jamming and facilitates lubrication with synovial fluid [39]. Recently Renner and co-workers [40] performed a retrieval analysis of BHR cups (Smith & Nephew, Memphis, TN, USA) and compared the clearance between 20 BHR shells, 31 MDM poly inserts and 24 ADM acetabular components of different sizes, and compared the clearance with both ADM and MDM inserts (MDM X3, Stryker, Mahwah, NJ, USA). Only 30.9 % of the MDM/BHR clearances were within the range of the MDM/ADM bearing. The authors’ studies suggest that the risk and benefits need to be evaluated on an individual basis and regular follow-ups are suggested to monitor for increased plastic wear. The study of Underwood et al. [41] had found a difference in terms of failure between the Articular Surface Replacement hip resurfacing arthroplasty of the DePuy ASR^TM^ and the BHR, asserting that the increased occurrence of edge loading in metal-on-metal hips augmented the design of the implant played a role. These observations in turn could validate the hypothesis that the absence of clearance conformity between DM liner and MoM cup could have played an important role in the early failure of the six cases reported in this review. The appropriate orientation of cup is very important in abduction and anteversion. Unfortunately, radiological data were missing in many cases or were not accurate. However, in five of the six IPD cases the abduction angles were beyond 45 degrees: from 48 to 72 degrees as reported by the authors, not taking into account the lowering of the coverage angle.

Finally, the few studies examining metal-on-metal (MoM) total hip implants, in which a comprehensive analysis of retrieved components has been performed [42,43,44], have clearly demonstrated the surface damage of the cup and the head of the implant, which was strongly correlated with blood metal ion levels, caused by a mal-positioned cup and a low clearance. Gascoigne and co-workers [45] studied articular surface damage of in situ monoblock BHR cups and long-term wear of a polyethylene DM liner in the case of mating these implants. In this study, thirty retrieved BHR monoblock cups, with a mean cup inclination of 47.3 degrees (SD 10.5) and version of 11.5 degrees (SD 7.6) were visually scored for damage features and areas of coverage. Visible damage on the BHR cups was primarily scratching and grooving of the surface, with a mean total damage score of 9.4 (SD 1.7) out of a possible 50; the damage score ranged from 5.0 to 11.7. They found generally mild damage and the average surface roughness of the retrieved Birmingham cups was considered low, suggesting an expected 10%–20% increase in DM prosthesis wear. However, they cautioned about the use of this technique in younger and more active patients because a higher-than-expected wear could be encountered when the monoblock cup is retained and articulated with a DM construct.

This review shows several limitations. First of all, some studies included are randomized controlled trials, but there are only six retrospective case series and several case reports. Moreover, the methodological quality is low because the series considered short-term follow-up where much relevant data, such as radiographic evaluation, were missed. Finally, only a few studies reported features about the type of femoral stems, which could be considered an important issue as a contributor to IPD. Finally, the follow-up period is too short to exclude possible further worsening of the results in the future. On the other hand, if we consider the incidence of 4.6% of IPD, the importance of follow-up is even higher that observed by Philippot et al. [46] who identified 81 cases (80 patients) with IPD from among 1960 primary THAs performed between January 1985 and December 1998, with a rate of 4.1%. This important difference of results should be theoretically more important. The mean time of occurrence of IPD in that series was 8.5 years (range, 1–15 years) with the PROFIL^®^ stem and 9 years (range, 4–17 years) in case of coupling with a PF stem. Both of these stems have been considered as “not friendly” [45,46,47,48]. In fact, the PROFIL^®^ was a modular, titanium stem with a rough 13-mm diameter neck, the PF^®^ was also a modular stainless steel stem with a 16-mm neck. The last consideration concerns the mobile inserts that were manufactured from ultra high molecular weight polyethylene (UHMWPE) sterilized in air. In 2009, a multicenter study from Massin et al. reported a lower incidence of IPD (0.3% at 10 years follow-up) [49]. The latest study with contemporary DM (implanted after 2000) reported no IPD at all [50,51,52,53].

## 5. Conclusions

In conclusion, according to the literature analyzed, we can stress that the concerns and doubts about mating a DM bearing with a large MoM cup cannot be dismissed. It has been pointed out that a DM bearing is not designed for an MoM cup; it is not mechanically tested on MoM cups, which presents different clearance and coverage angles. Predictable complications may occur, such as IPD, polyethylene wear and true dislocation. These complications have been reported at a rate even higher than that which occurred in the eighties, when the first generation of DM implants were of a lower quality of polyethylene and the characteristic of the design was not as optimal as the modern ones. Future developments in this direction, together with in vitro studies, could be useful to carry out remarkable discussions on comparable results and acquire a better knowledge about this matter.

## Figures and Tables

**Table 1:** The scientific literature that meets our criterion.

Author	MoM Type	MoM+ DM Cup + Liner	Off Label (Yes-No)	Complication Failure/Time	FU (Months)	Average Cup Abduction/Anteversion
Kasparek et al. 2018 [13] Case series 11 revisions	11 MoM-THAs	BHR (Smith & Nephew) + ADM/MDM^®^ X3 (Stryker)	Y	Group 1: 1 (7 days) Group 2: 0 Group 3: 1 (Clicking noise)	31 (24–37)	Not reported
McPherson and Sherif 2014 [15] Case report revision	1 MoM-THA+ cementless triflange porous pelvic implant	M2a-Magnum(Biomet) + Active Articulation DM E1-(Biomet)	N	Group 1: 1 (1 month) + dislodgment Group 2: 0 Group 3: 0	1	Not reported
Samona et al. 2016 [23] Case report revision	1 MoM-THA	Cup Biomet not mentioned + Active articulation DM E1(Biomet)	N	Group 1: 0 Group 2: 1 + poly wear (within 12 months) Group 3: 0	1	Not reported
Brazier et al. 2018 [24] Case report revision	1 MoM-THA	M2a-Magnum(Biomet) + Active Articulation DM E1^®^ (Biomet)	Y	Group 1: 0 Group 2: 1 + poly wear 12 months Group 3: 0	1	72°
Riviere et al. 2013 [17] Case report revision	1 MoM-HRA	BHR (Smith & Nephew) + ADM/MDM X3 (Stryker)	Y	Group 1: 0 Group 2: 1 + poly wear 14 months Group 3: 0	14	52°
Plummer et al. 2016 [20] Case series 25 revisions (14 MoMs)	11 MoM-HRAs 14 MoM-THAs	BHR (Smith & Nephew)/M2a-Magnum(Biomet)/Wright Corserve + Liner not mentioned	Y	Group 1: 0 Group 2: 1 + poly wear 19 months Group 3: 0	29 (24–45)	49°(39°–67°)
Blevins et al. 2019 [25] 71 revisions retrospective cohort series	27 MoM-HRAs	BHR + ADM/MDM (Stryker)	Y	Group 1: 1 (1 month) Group 2: 2 (3.8–3.9 years) Group 3: 0	17	48.7°(37.9°–59.0°)/ 24°(10.4°–42.8°) In case of IPD 50°–48°/ 27°–43°
Colacchio et al. 2019 [26] 143 revisions	19 MoM-THAs 10 MoM-HRAs	19 M2a-Magnum (Biomet) + 9 BHR (Smith & Nephew) + 1 Wright BFH Technology associated with 19 Active articulation DM E1 (Biomet) + 10 ADM/MDM (Stryker)	Y	Group 1: 1 (1 month) Group 2: 0 Group 3: 1 periprosthethic fracture	47 months (24–62)	<60°
Pritchett et al. 2014 [18] Case series 14 revisions	14 MoM-HRAs	BHR (Smith & Nephew)/ASR-Recap-Durom (Biomet)/M2a-Magnum^®^ (Biomet)/Wright Conserve + liner not mentioned	Y	Group 1: 0 Group 2: 0 Group 3: 0	41 (36–53)	30°–60°
Sassoon et al. 2016 [21] Case report revision	1 MoM-THA	BHR (Smith & Nephew) + ADM/MDM X3^®^ (Stryker)	Y	Group 1: 0 Group 2: 0 Group 3: 0	14	Not reported
Verhelst et al. *2012* [16] Case series 3HRAs revisions	3 MoM-HRAs	2 Recap (Biomet)/1 BHR (Smith & Nephew) + Avantage Active (Biomet)	Y	Group 1: 0 Group 2: 0 Group 3: 0	6	52°–54°
Figueras et al. 2016 [22] Case series 10 revisions	2 MoM-HRAs 8 MoM-THAs	Not mentioned	Y	Group 1: 0 Group 2: 0 Group 3: 1 superficial infection	25.6 (6–45)	48.9°(38°–56°)
Snir et al. 2015 [19] 18 revisions	6 MoM-HRAs + 3 MoM-THAs	cup non mentioned + ADM/MDM X3^®^ (Stryker) and Active Articulation DM E1-(Biomet)	Y/N?	Group 1: 0 Group 2: 0 Group 3: 0	22(6–45)	39.2°

HRA: Hip Resurfacing Arthroplasty; MOM-THA: Metal-on-metal Total Hip Arthroplasty.

**Table 2:** Group 1: True dislocation; Group 2: IPD (Intra-prosthetic dislocation); Group 3: other complications.

Complications	Number of Complications	Rate (%)
Group 1 (True dislocation)	4	3.05%
Group 2 (Intra-prosthetic dislocation IPD)	6	4.6%
Group 3 (Others complications)	4	3.05%
Total	14	10.7%
